# Tokophobia: Case Reports and Narratives of Ten Japanese Women

**DOI:** 10.3390/healthcare11050696

**Published:** 2023-02-26

**Authors:** Mizuki Takegata, Yuriko Usui, Satoshi Sohda, Satoru Takeda, Jun Takeda, Tomomi Saito, Yasuyo Kasai, Hideki Watanabe, Megumi Haruna, Yukiko Ohashi, Toshinori Kitamura

**Affiliations:** 1 Kitamura Institute of Mental Health Tokyo, Tokyo 151-0063, Japan; 2 Department of Midwifery and Women’s Health, The Graduate School of Medicine, The University of Tokyo, Tokyo 113-8654, Japan; 3 Department of Obstetrics and Gynecology, Graduate School of Medical Science, University of Tsukuba, Tsukuba 305-8577, Japan; 4 Department of Obstetrics and Gynecology, Juntendo University, Tokyo 113-8421, Japan; 5 Aiiku Research Institute for Maternal, Child Health and Welfare Imperial Gift Foundation Boshi-Aiiku-Kai, Tokyo 106-0047, Japan; 6 Department of Perinatal Mental Health of Aiiku Clinic, Aiiku Maternal and Child Health Center, Tokyo 105-8321, Japan; 7 Japanese Red Cross Medical Center, Tokyo 150-8935, Japan; 8 Nagato Clinic, Tokyo 120-0002, Japan; 9 Faculty of Nursing, Josai International University, Chiba 283-0002, Japan; 10 Kitamura KOKORO Clinic Mental Health, Tokyo 151-0063, Japan; 11 T. and F. Kitamura Foundation for Mental Health Research and Skill Advancement, Tokyo 151-0063, Japan; 12 Department of Psychiatry, Graduate School of Medicine, Nagoya University, Nagoya 464-8601, Japan

**Keywords:** tokophobia, lived experience, qualitative study, Japanese women

## Abstract

Intense fear of childbirth by expectant women is called tokophobia. Because there are no qualitative studies targeting women with an intense fear of childbirth in Japan, it is unknown whether there is any link between the type of fear of objects/situations among tokophobic women and their psychological/demographic background. Furthermore, there is no available summary of the lived experience of Japanese women with tokophobia. This study aims to identify the intensity patterns of various types of fear among the participants and summarize the lived experience of having intense fear of childbirth. A qualitative descriptive study was conducted using a semi-structured interview. Pregnant women with an intense fear of childbirth participated in individual interviews facilitated by a psychiatrist and a midwife. Audio recordings of the interviews were transcribed and analyzed using a content analysis approach. The number of participants was ten. The types of feared objects varied individually and these were categorized as being related to either prospective or retrospective fear. The participants’ experiences were grouped into three categories: difficulty in daily life, preoccupied negative expectation towards childbirth, and psychological adaptation to the upcoming childbirth. The results imply that women with tokophobia continuously suffer from fear in their daily life; hence, a special approach is needed to detect and reduce their fear.

## 1. Introduction

When a woman becomes pregnant, she may have both positive and negative feelings towards her pregnancy and childbirth; childbirth may be a pleasant event welcoming a new family member. On the other hand, pregnant women be afraid of the upcoming childbirth and concerned about a variety of aspects, such as labor pain, medical intervention, and/or a deviant course of labor [1,2,3,4,5]. Having such ambivalent feelings is natural during the psychological adaptation to their new life. Fear of childbirth itself can be seen in the majority of pregnant women in a clinical setting. Other pregnant women may dread childbirth, which is called tokophobia [6,7]. Tokophobia is a pathological fear of childbirth which may lead to the avoidance of pregnancy. The prevalence of tokophobia is estimated to be 14% [8]. Intense fear of childbirth is associated with enduring anxiety and difficulties in daily life [9]. Women with an intense fear of childbirth are more likely to describe their childbirth as painful and traumatic [10,11]. Women with an intense fear of childbirth tend to choose caesarean sections (CS) even when there is no clear medical necessity for the procedure [6,12,13,14].

It is generally recognized that there are two types of tokophobia, according to the history of pregnancy/childbirth: primary and secondary. Primary tokophobia is the intense fear of childbirth held mainly by nulliparous women [5,6]. It is the fear of the unknown because childbirth has not been experienced before [6,7], which is also called a prospective phobia [5]. Secondary tokophobia is what is felt by multiparous women who have experienced traumatic childbirth [6,7]. Fisher et al. (2006) [5] termed it retrospective phobia.

There are several qualitative studies exploring the subjects for the fear of childbirth [2,3,5,15,16,17]. They demonstrated that fear objects/situations are multi-faceted: pain, unpredictable complications such as bleeding, and uncontrollability [6,17,18]. Takegata et al. (2018) [4] firstly conducted a focus group interview to explore the subjects of the fear among pregnant Japanese women with a low to moderate fear of childbirth [4]. This study showed that the root of the fear that Japanese women expressed was similar to that expressed by women in previous studies [2,3,5,6,15,17,18]. Unlike the previous reports, fewer Japanese participants opted for elective CS regardless of the degree of their fear because of their concerns of postoperative risk, pain, and delayed bonding with the child [4].

However, there has been no qualitative study targeted at tokophobic women with an *intense* fear of childbirth in Japan. Although it is known that the fear objects/situations that women with mild and moderate fear of childbirth have can be multiple, it is not known whether tokophobic women can manifest a *specific* fear object/situation (either fear of labor pain, fear of child-risk, or another) or, alternatively, if they can manifest fear toward all kinds of objects/situations intensively, or whether the type of fear objects/situations is influenced by their psychological background.

Our aims for the present study are (a) to determine whether there is any link between the type of fear objects/situations among tokophobic women and their psychological/demographic background, and (b) to summarize the lived experience of fear of childbirth among Japanese women with tokophobia. Summarizing lived experiences of the fear of childbirth among Japanese women with tokophobia may also be important for considering clinical implications for health professionals in the context of the medical culture in Japan. Detailed psychopathological and nosological discussions based on the same sample will be reported elsewhere.

## 2. Methods

### 2.1. Design, Participants and Procedure

This qualitative study was part of a main study aimed at reconsidering the psychiatric diagnosis of tokophobia through qualitative exploration (unpublished). The interview guide is a semi-structured one with particular attention to the psychopathological (phenomenological) aspect of tokophobia. In addition, this interview allowed participants to talk freely about their fear and anxiety and explain the feelings they experienced toward upcoming childbirth. These accounts from tokophobic women will be summarized in this study because it is important for clinical professionals to understand their perspective fully.

Pregnant women attending one of the four collaborative obstetric health facilities in Tokyo were invited to participate in this qualitative study if they were identified as having a severe fear of childbirth by the responsible obstetrician. The recruitment period was between December 2019 and February 2021. The four obstetric health facilities were located in different areas of central Tokyo: two were tertiary level hospitals, one was a clinic which only provides antenatal service, and the remaining clinic has outpatient and inpatient departments. The eligible criteria were any pregnant women who could converse in Japanese, who had manifested an intense fear of childbirth, and were over the age of 20. Obstetricians identified women with a severe fear of childbirth *from their clinical perspectives* rather than asking mothers to complete screening questionnaires for assessing fear of childbirth because it was more feasible and accurate in the busy outpatient departments. If the obstetricians were unsure about identifying a woman with an intense fear of childbirth, it was suggested that they use the Visual Analogue Scale (VAS) by asking the mother “How much are you afraid of childbirth? (0: not at all, 100: extremely afraid of childbirth)” to save time.

Eligible women identified at each facility were solicited to participate in this study by an obstetrician who showed them the invitation brochure. When they agreed, they were asked to contact either MT or TK by email or phone. An informed consent form and the Japanese Wijima Delivery Expectancy/Experience Questionnaire (WDEQ [19]) version A [20], which was used to assess the antenatal fear of childbirth, were sent to the participant’s house via post a few days prior to the interview day. The informed consent form with the signature of agreement as well as the completed JWDEQ version A were given to the researcher on the interview day (before the interview started). A qualitative, descriptive design with individual interviews was used. Although in-person interviews were conducted at the beginning of the study, the COVID-19 pandemic, which started in January 2020, meant that we converted our interview to online methods. For the online interviews, the signed informed consent form and the completed JWDEQ version A were sent back to the Kitamura Institute of Mental Health Tokyo by postal service prior to the interview date. The one-hour interview was conducted by TK (psychiatrist) with support from MT (midwife). During the interview, the participant was asked to answer how afraid she was of each of the following fear objects/situations using a 5-point Likert scale (1 being not at all to 5 being very much): pain, life risk of mother, life risk of child, and uncontrollable situation, which were depicted based on the previous study interviewing Japanese pregnant women with a mild to moderate fear of childbirth (Takegata et al. 2018) [4]. Answers of 1 and 2 were categorized as low, 3 as middle, and 4 and 5 as high. In addition, each participant was asked to explain more about the object/situation of fear (pain, life risk of mother, life risk of child, and uncontrollable situation) in detail. Throughout the interview, participants spontaneously expressed how they ‘experienced’ the fear of childbirth without being guided by the interviewer. In addition, acceptance of pregnancy (Q. how did you feel when you found you were pregnant?) and talking to fetus (Q. how often do you talk to your unborn child?) were asked to the participants. Answers of 1 and 2 were categorized as low, 3 as middle, and 4 and 5 as high.

Each participant was given a temporary English name to assure anonymity while the whole conversation was audio recorded.

### 2.2. Analysis

The audio recordings of the interviews (with permission of the participants) were transcribed. The verbal statements related to *object*/*situation of fear*, were coded by unit, and cross-checked if there was an additional theme of fear in the verbal statements. On the other hand, for the narratives of the participants related to the experience of fear of childbirth, the verbal statements were coded by unit, then codified in vivo, subcategorized, and categorized by the first author (MT) following the content analysis methods [21]. The second author (YU) carefully reviewed the categories and wordings.

### 2.3. Ethical Assurance

Ethical approval for the present study was obtained from the Ethics Committee of the Kitamura Institute of Mental Health Tokyo, Japan (no. 2019090801). Voluntary participation was ensured by using the informed consent sheet and providing verbal explanations. All electronic and paper-based information collected was carefully stored in a secure location by TK to ensure confidentiality.

## 3. Results

Ten pregnant women participated in this qualitative study (Table 1). Most of the participants were nulliparous (*n* = 9). One participant was in the first trimester, two were in the second trimester, and seven were in the third trimester. Overall, the JWDEQ scores of the participants for assessing fear of childbirth were higher than 70.

### 3.1. Objects/Situations of Fear

As shown in Table 2, intensities and contents of fear of childbirth (pain, medical intervention, risk of child, risk of mother, being in a panic state, and being isolated) varied among the participants. Because the participants were asked to explain more about each content of fear, there were no other contents of fear depicted in this study.

#### 3.1.1. Participants Who Are Mostly Afraid of Labor Pain, with or without Medical Intervention

Six participants mentioned that they were most afraid of pain (ID 2, 3, 5, 6, 7, and 10).

Erika (ID2), who was at 37 gestational weeks, had experienced three miscarriages. She had been to a psychiatric clinic for panic disorder when she was 28 years old, but she was not having any reoccurrence of symptoms during pregnancy at the time. She was most afraid of receiving an epidural analgesia (together with labor pain) due to the experience of having had miscarriages. Having focused only on becoming pregnant, she had never thought about labor pain realistically until she became pregnant and then her fear started. Even though obstetric analgesia was attractive to her (reduction of pain), she refrained from receiving the service because she was very afraid of the injection.

*“I never thought of my fear of childbirth or labor pain because I just focused on getting pregnant. But after I got pregnant, childbirth became more realistic to me, and I started to feel frightened.”*

Alice (ID3), who was at 32 gestational weeks, mentioned that her fear was the most intense during her first trimester when she suffered from severe hyperemesis. She could not stop browsing relevant information about pregnancy, childbirth, and health facilities. When she read terrifying stories about childbirth shared by others on the internet, her fear rapidly escalated. Although her mental state had become calm when she recovered from morning sickness, she was still moderately afraid of childbirth and this fluctuated to an intense level. Although labor pain was her major concern, she decided to give birth at an obstetric clinic which provided no obstetric analgesia because there was no such obstetric facility offering obstetric analgesia in her hometown.

*“Labor pain was totally uncertain to me. Most of my friends said labor pain was extremely painful, unlike anything they had ever experienced before.”*

Sara (ID5), who was at 31 gestational weeks, once attended a psychiatric clinic due to an overdose of subscribed medicine at the age of 32. It was when she entered university (at around 19 years of age) that she started to think that childbirth was totally unsafe, painful, and frightening even though her image of childbirth was vague. Such fear became much stronger during the current pregnancy when she heard negative childbirth stories from others. In addition to the fear of labor pain, she felt extremely uneasy to see health professionals wearing white gowns or when being exposed to a hospital environment. She mentioned that her blood pressure always increased while in the hospital. She was suffering from fibromyalgia and was afraid of “all kinds of medical interventions”.

*“I feel frightened when I see health professionals wearing white jackets. I also don’t like the atmosphere of the hospital and its smell.”*

Alicia (ID6), who was at 38 gestational weeks, with no experience of psychiatric treatment, was afraid of all kinds of medical interventions as well as labor pain. She tried to avoid thinking about childbirth, however her fear spontaneously arose resulting in her spending 80% of the day dreading childbirth. She decided to receive obstetric analgesia; however, she mentioned the decision was only slightly effective in reducing her fear. She mentioned that her heart always began to beat rapidly when she visited an outpatient department.

*“I feel relieved to some extent by choosing analgesia. However, I am still wondering if it is really helpful.”*

Stephanie (ID7), who was at 27 gestational weeks, had an experience of receiving psychological counselling for three years due to a maladaptive interpersonal relationship. Having become pregnant by in vitro fertilization (IVF), she was determined to receive obstetric analgesia. However, she was still anxious about labor pain but not medical intervention.

*“Before pregnancy I tried not to think about childbirth realistically. But I started to feel afraid of labor pain because my friends told me the pain will not be reduced 100% even with obstetric analgesia. So, I am still trying not to think about labor pain.”*

Anna (ID8), who was at 35 gestational weeks of pregnancy, had an experience of receiving cervical conization. Hence, she was very afraid of medical interventions, especially injections or epidural analgesia. Whenever she was injected, her blood pressure increased abruptly.

*“My heart starts pounding whenever I get an injection in a dentist’s office since I was a small kid. When I got the surgical operation for cervical cancer, I was most afraid of the anesthesia rather than the surgical operation itself. I felt I might not be able to wake up again.”*

Scarlet (ID10), who was at 12 gestational weeks of pregnancy, scored nearly the maximum number of points on JWDEQ version A (164). Although she did not take any contraceptives after her marriage, she suddenly got panicky during her first visit to the clinic when her pregnancy was confirmed. She cried in front of the obstetrician and regretted becoming pregnant. She decided to receive obstetric analgesia; however, she was concerned as to whether obstetric analgesia would really work in childbirth. On the other hand, she usually avoided visiting health facilities in her life.

*“(Why are you afraid of labor pain?)… Why? Because I feel like I will not be able to endure the pain, I may become insane. I know that obstetric analgesia can reduce labor pain, however, I am concerned that there might be the case that the anesthesiologist may be too busy to offer pain relief to me, or if the pain relief would not work… that makes me so anxious.”*

#### 3.1.2. Participants Who Were Mostly Afraid of Life Risk of Mothers and Children

Jessica (ID1), who was the only multiparous woman in this study, received obstetric analgesia during her previous childbirth. She decided to give birth at a facility with no obstetric analgesia service this time because the facility was closer to her house allowing her to care for her first child. Therefore, fear of pain was one of her great concerns about the upcoming childbirth.

*“As I mentioned before, my first baby got stuck even though I received medication to induced labor. I was suffering from weak labor for one week. But the induced labor was extremely painful…”*

More than fear of labor pain, she was very much afraid of the situation that her unborn child may be at risk of life due to her older age and uncertain situation of birth (fear of life risk). Another concern of hers was that she might be separated from her partner during her childbirth (fear of isolation) because she hesitated to tell the hospital staff that her partner was a woman.

*“I remembered that I was concerned for my baby so much during my first delivery. It was a prolonged labor. I was very scared if he might get weak.”*

Angelina (ID9) experienced several miscarriages despite having infertility treatments for more than 5 years. She was devastated by the experiences of the miscarriages. She became pregnant over the age of 40 and thought that her pregnancy continuing to the last trimester was “miraculous”. She was extremely afraid about still birth and all the kinds of risks for the unborn child during childbirth (fear of life risk).

*“My husband and I have been very skeptical about being optimistic and expecting the situation in positive way… (cry). But my positive emotion looking forward to meeting my unborn baby comes up spontaneously… (cry). For example, expecting that I will play with the baby in somewhere… (cry). But, I am very fearful if the baby will die … (cry).”*

#### 3.1.3. Participant Who Was Afraid of Medical Intervention, Panicky State, and Risk of Mother

Rosaline (ID4) experienced sexual assault in childhood. She left her house as an adolescent and lived by herself by hiding her real age. She had depression and post-traumatic stress disorder (PTSD) with several occasions of parasuicide until she met her current partner. She always fainted and/or fell into a panicky state when she was faced with receiving an “internal examination” (fear of medical intervention). She would start having difficulty breathing when she imagined being on the delivery table (fear of risk of mother). She strongly believed that she would die on the date of delivery due to her panicky attitude and being exposed to unendurable labor pains, hence she wrote a will believing that she would die during childbirth.

*“I understand there is low possibility of dying due to childbirth in the modern society. However, my emotions and belief that I would die comes up suddenly and this is different from my rational understanding. Unexpectedly, I felt like I would die on the day of childbirth and tried to make a will.”*

### 3.2. Experience of Fear of Childbirth

The participants’ lived experiences of fear of childbirth can be divided into three groups: (a) difficulty in daily life, (b) preoccupied negative expectation towards childbirth, and (c) psychological adaption to upcoming childbirth (Table 3).

#### 3.2.1. Difficulty in Daily Life

All of our participants claimed to have experienced difficulty in daily life due to tokophobia. The difficulty in daily life category consists of two subcategories: *preoccupied fear of childbirth at every moment* and *reduced social activities*. Most of the participants were preoccupied with fear which would suddenly come up in their minds. Fear repeatedly occurred not only during the daytime but also at night-time. Their fear was ruminative even though they recognized the feelings as irrational. Such fear was sometimes/often so uncontrollable that some of the participants expressed difficulty doing house chores or concentrating on work. They even avoided meeting close friends.

*“I spend most of my time searching for information about childbirth on websites during the weekend. I often get irritated when being called by my sister. I tried not to meet a close friend because of my mental state.”*

(Scarlet, ID10)

#### 3.2.2. Preoccupied Negative Expectation towards the Childbirth

This category consists of five subcategories: expecting the worst situation during childbirth, difficulty of reducing fear of childbirth even with indisputable evidence, loss of control due to uncertainty, lack of positive image of childbirth, mixed feelings with positive and negative thoughts, and sense of losing identity. Many participants showed strong resistance to viewing childbirth in a more optimistic way. Some of the participants admitted they had a strong (but erroneous) belief that their childbirth would be worse than normal. Even if the hospital staff said “the possibility of a bad scenario is one out of 100 or 1000 deliveries”, these women believed that they themselves would be the one. In addition, even though they admitted that having intense fear was irrational, it could never be denied or ameliorated. There were other women who even believed that their intense fear was quite natural and other people’s perspectives were nonsense. Most of the participants felt loss of control due to uncertainty and because nothing could be done in advance. For them, childbirth was too uncertain to escape. Moreover, they never understood why and how other women were looking forward to the childbirth. Alternatively, they had mixed feelings with positive and negative thoughts. Some of the participants even had a sense of losing their identity. They felt that they were different from how they were before pregnancy.

#### 3.2.3. Psychological Adaptation to Upcoming Childbirth

Of interest was the notion stated by one participant (ID7, Stephanie) that helped her with regard to childbirth. She mentioned that she would have to *accept* childbirth as it is because she understood that many women might feel fear of their upcoming childbirth as she did. She tried not to think too much about her childbirth.

*“I am very afraid of childbirth, but I see many other women like me would also feel intense fear for childbirth, so, I am one of them… I feel terrified, but I also feel there is nothing to escape... so I am trying to feel myself as empty. All I have to is to accept the situation.”*

(Stephanie)

## 4. Discussion

This qualitative study targeted pregnant women suffering from *severe* fear of childbirth in Japan, and it aimed to obtain a clear picture of tokophobia in order to fully understand women who suffer from it. To our knowledge, this is the first qualitative study that has targeted tokophobic women in the East Asian region.

### 4.1. Objects/Situations of Fear

Our study identified links between the fear objects/situations among participants and their backgrounds. “Primary tokophobia” generally indicates fear shared by *nulliparous* women whereas “secondary tokophobia” affected *multiparous* women because of their previous negative birth experiences [5,6]. Fisher also categorized primary tokophobia as prospective fear caused by uncertainty and lack of experience and secondary tokophobia as retrospective, which was associated with PTSD. However, the results of our qualitative study indicated a more complex scenario. The distinction between prospective and retrospective tokophobia may be better viewed in terms of the content of the fear in individual cases rather than dividing the types of tokophobia by birth parity (nulliparas vs. multiparas). Secondary tokophobia is better categorized as cases of women who have experienced miscarriage, still birth, sexual abuse, and other traumatic experiences regardless of their parity.

#### 4.1.1. Fear of Labor Pain among Primiparous Women

Our study identified that pain was one of the major concerns for most of the nulliparous participants. However, fear of labor pain was comorbid with fear of medical intervention among some of the participants whereas others were afraid of either labor pain or medical intervention. It was of note that nulliparous participants were mostly concerned about labor pain when they heard unpleasant stories from others in this study. The image of childbirth among nulliparous women was strongly influenced by social contexts such as listening to unpleasant stories of others, TV, and other social media [5].

In addition, some of the nulliparous participants mentioned that the fear was not as strong before pregnancy; hence, they focused on getting pregnant. Hofberg and Brockington (2000) noted that the women with primary tokophobia were likely to have had intense fear in adolescence and actively avoided pregnancy due to the fear of childbirth whereas others planned to get pregnant despite their fear. We are unaware of the size of the female population who have such an intense fear of childbirth that they avoid pregnancy in Japan. However, because having a child is generally one of the important social events, not only for a woman but also her partner and family members, participants might have suppressed their fear consciously or unconsciously until pregnancy. Further study is needed to investigate the relationship between antenatal fear of childbirth and avoidance of pregnancy, quantitatively. Another important issue that remains to be studied is the personality traits of tokophobic women [22]. Of particular interest is excessive sensitivity to pain in general.

#### 4.1.2. Fear of Medical Intervention and Life Risk of Mother and Baby

The participants who were afraid of medical interventions as well as the risk to the mother and baby had past traumatic experiences (though not necessarily fulfilling the criteria of PTSD), such as receiving a surgical treatment, being sexually assaulted, and repeated miscarriages. It is of note that fear of medical intervention is not always accompanied by fear of pain. Some were concerned about the side effects of medical intervention such as analgesia, and some others dreaded needle injections (needle phobia). Fear increased in some participants when they were in a hospital setting or were exposed to health professionals wearing white gowns (white-coat hypertension). These phobias, such as needle phobia or white-coat hypertension, are categorized under the specific phobia of DSM-5. Rosaline (ID4) showed extreme fear of internal examinations due to her experience of being sexually assaulted. Her symptoms were more akin to PTSD than a specific phobia. Her symptoms were different from needle phobia and white-coat hypertension. It is of clinical importance for a health professional to know about patients’ past surgical operations or other experiences in clinical settings. However, special attention should be paid when asking women about these experiences so that they do not feel offended or emotionally hurt when disclosing traumatic experiences.

In line with a previous study [9], our participants were suffering from *difficulties doing daily activities* such as being unable to concentrate on work and house chores, or even avoiding meeting friends and difficulty with communication. Being unable to control fear was another central point that was consistent with other qualitative studies [2,3]. Our participants talked about unexpected and sudden bursts of fear in their daily life, which were hard to supress or be distracted from. Additionally, some participants could not stop searching for relevant information on the internet, which led to adverse effects on their mental states. There might be interactions between ruminative ideas and behavior addiction. Although this study did not investigate vulnerable personalities associated with anxiety or depression, such ruminative ideas and addictive behavior may be a major factor that contributes to the deteriorating mental states of women.

The subcategory of *expecting the worst situation during childbirth* was newly identified compared with other qualitative studies targeting pregnant women with mild or moderate fear of childbirth who expressed ambivalent feelings to forthcoming childbirth [4,16]. Some of the present participants showed resistance to having positive expectations towards childbirth. Whenever a positive emotion arose spontaneously, they tried to supress it: “I should not expect positive things”. Such attitudes may be a way of providing self-protection against situations where they could be disappointed, in which their emotional conflict would be very intense. In addition, the participants found it difficult to reduce their fear through cognitive understanding that the worst situations in childbirth are a very low possibility or by receiving sufficient medical knowledge about childbirth. Lack of antenatal education is often noted as a significant factor that affects fear and worry in pregnant women [16,23,24], but the effect of antenatal education on reducing the fear of childbirth is still uncertain [23,24]. Providing proper education for pregnancy and childbirth may only be partially effective and possibly only among a specific group of women. Different approaches should be considered in order to support women in coping with their fear.

### 4.2. Clinical Implications

A variety of approaches are required for women with a general fear of childbirth and for those with tokophobia. First, as mentioned as above, it is of importance for perinatal health professionals (obstetricians and midwives) to *be aware of* women with tokophobia. Presumably, fear of childbirth has not been broadly recognized by health professionals in clinical settings, not only in Japan but also in several countries in Asia. Fear of childbirth is often considered as “normal” among perinatal health care professionals, hence there is a failure to detect tokophobic women. Therefore, increasing awareness and understanding of the fear of childbirth should be a priority so that perinatal health professionals can detect women who have a severe fear of childbirth. Special caution should be exercised and effective interview skills should be used when assessing tokophobia because societal norms define pregnancy and childbirth as delightful; therefore, expectant women would likely hesitate to disclose the fear that they have.

Second, although a standard intervention approach has not been established, creating a comprehensive support system that ensures a safe and comfortable environment, which includes continuous counselling during the antenatal period and building trust with health professionals in outpatient settings and the delivery room, is essential [25]. In addition, assessment will be needed to understand an individual’s fear by discriminating which element is “prospective fear” or “retrospective fear” through frequent and effective communications. As shown in our study, tokophobia is often linked to past traumatic events and possibly the woman’s personality, as such, professional support should go beyond a simple reassurance (e.g., “It’s all right. Everything will go smoothly.”) to provide deeper psychological support including cognitive therapy, interpersonal therapy, and brief dynamic psychotherapy.

Third, a strong link between intense fear of childbirth and bonding disorders was not confirmed by this qualitative study due to the limited number of participants. However, some of the participants desired having a child and frequently talked to the fetus during pregnancy. Therefore, paying more attention to this link, or conducting a further study is required to identify what characteristics/experiences of women are associated with bonding disorders.

## 5. Conclusions

This qualitative study surveyed ten pregnant Japanese women with an intense fear of childbirth and aimed to identify the source of their fear and the lived experience of women with tokophobia, qualitatively. The multi-aspect fear concerns identified by the participants were categorized as either prospective or retrospective fears that were linked with parity and negative experiences in the past, leading to complicated symptoms. Symptoms differ from one woman to another. Furthermore, the results of the qualitative analysis of their experiences showed that there were three main categories: difficulty in daily life, preoccupied negative expectations towards childbirth, and psychological adaptation to upcoming childbirth. The results implied that women with a fear of childbirth were continuously suffering from fear in their daily life; hence, a tailor-made approach is needed to detect and reduce their fear.

## Figures and Tables

**Table 1:** Participants’ characteristics.

ID (Name)	Age	Birth Parity	Experience of Miscarriage	ART	Gestation Weeks	Employment	Preference of Obstetric Analgesia	Remarks of Past Experience
1.Jessica	40	Multipara	-	AID	22	Employed	No	
2.Erika	37	Nullipara	3 times	-	33	Employed	No	Experience visiting a psychiatric clinic due to panic disorder at the age of 28
3.Alice	30	Nullipara	-	-	32	Employed	No	Experienced hyperemesis gravidarum at the early stage of pregnancy
4.Rosaline	35	Nullipara	-	AID	36	Housewife	Yes	Experience of child abuse
5.Sara	35	Nullipara	-	AI	31	Employed	Yes	Fibromyalgia (at the age of 25)/Experience visiting a psychiatric clinic due to suicidal ideation at the age of 32
6.Alicia	33	Nullipara	-	-	38	Employed	Yes	
7.Stephnie	31	Nullipara	-	IVF	27	Employed	Yes	Experience receiving psychological counselling at the age of 28
8.Anna	35	Nullipara	-	-	35	Employed	No	Cervical conization surgery at the age of 32
9.Angelina	42	Nullipara	>3 times	IVF	35	Employed	No	Infertility treatment over 5 years
10.Scarlet	34	Nullipara	-	-	12	Employed	Yes	

AID: Artificial Insemination by Donor. AI: Artificial Insemination. IVF: In Vitro Fertilization.

**Table 2:** Intensity and content of fear of childbirth among participants.

ID	Japanese W-DEQ	Subject of Fear of Childbirth						Acceptance of Pregnancy	Talking to Fetus	Remark
		Pain	Medical Intervention	Risk of Child	Risk of Mother	Being in a Panic State	Being Isolated			
1	104	High	Low-Middle	High *	High	Low	High	Positive	Frequent	
			(Injection)							
2	79	High *	High	Middle-Strong	Low	Low	-	Positive	Frequent	
			(Epidural analgesia)							
3	81	High *	Low-Middle	Middle	Low	Low	-	Positive	Frequent	
			(Injection)							
4	93	Middle	High *	Middle	Strong	High	-	Positive	Frequent	Becoming panicky for an internal examination. Wrote a will thinking she would die during childbirth.
			(Internal examination)							
5	134	High *	High	Middle-Strong	Middle-High	High	-	Was a little confused at the beginning	Unknown	White-coat hypertension
			(All kinds of medical interventions)							
6	105	High *	High	Strong	High	Middle	-	Was a little confused at the beginning	Frequent	
			(All kinds of medical interventions)							
7	119	High *	Low-Middle	Middle	Middle	Low	Middle	Not mentioned	Rare	Concerns if obstetric analgesia will work in childbirth.
			(Injection)							
8	83	High *	Low-Middle	Low	Middle	High	-	Positive	Sometimes	Having fear of labor pain but intensively afraid of obstetric analgesia.
			(Injection/Epidural)							
9	108	High	High	High *	Middle	Middle	-	Was a little confused at the beginning	Sometimes	Intensely afraid of still-birth, or anomaly of fetus
			(All kinds of medical interventions)							
10	164	High *	High	High	High	High	High	Was confused at the beginning	Unknown	Concerns if obstetric analgesia will work in childbirth. On the other hand, avoided visiting health facilities in her life
			(Injection)							

*: the subject a woman considers the most frightening.

**Table 3:** Experience of severe fear of childbirth.

Category	Sub-Category	Code
Difficulty in daily life	Psychological disturbance in daily life due to preoccupied fear of childbirth.	The image of childbirth is nothing but ‘fear’
		Rumination of negative thoughts and fear which emerges suddenly
		Uncontrollable fear
		Frequent recurrence of fear despite recognizing the feeling is totally irrational
	Reduced social activity	Difficulty doing house chores due to depressed mood
		Avoiding meeting others
		Difficulty in smooth communications with partner
Preoccupied negative expectation towards the childbirth	Expecting worst situation during the childbirth	Resistance to optimistic perspective
		Idea that situation will go to the worst scenario
	Difficulty reducing fear of childbirth even with indisputable evidence	Belief that the extreme fear is irrational, but can never be denied
		Belief that the extreme fear is totally natural and inevitable
	Loss of control due to uncertainty	Nothing can be done in advance
		Childbirth is too uncertain to escape
	Lack of positive image of childbirth	Envy women who look forward to childbirth
		Looking forward to the childbirth is totally nonsense
	Mixed feeling with positive and negative thoughts	Childbirth and fear of pain are different
		Mixed feeling of having joy to meet a baby and fear
	Sense of losing identity	I, who is afraid of childbirth is not I, who I was before pregnancy.
Psychological adaption to upcoming childbirth	Let myself go with the flow	Allow myself the natural flow of childbirth
		Let myself be ‘empty’

## Data Availability

Due to the nature of this research, participants of this study did not agree for their data to be shared publicly, so supporting data are not available.
